# Neonatal seizures alter NMDA glutamate receptor GluN2A and 3A subunit expression and function in hippocampal CA1 neurons

**DOI:** 10.3389/fncel.2015.00362

**Published:** 2015-09-23

**Authors:** Chengwen Zhou, Hongyu Sun, Peter M. Klein, Frances E. Jensen

**Affiliations:** ^1^Department of Neurology, Division of Neuroscience, Boston Children’s HospitalBoston, MA, USA; ^2^Program in Neurobiology, Harvard Medical SchoolBoston, MA, USA; ^3^Department of Neurology, Perelman School of Medicine, University of PennsylvaniaPhiladelphia, PA, USA

**Keywords:** GluN receptor subunit, post-seizure, development, immature brain, synaptic transmission

## Abstract

Neonatal seizures are commonly caused by hypoxic and/or ischemic injury during birth and can lead to long-term epilepsy and cognitive deficits. In a rodent hypoxic seizure (HS) model, we have previously demonstrated a critical role for seizure-induced enhancement of the AMPA subtype of glutamate receptor (GluA) in epileptogenesis and cognitive consequences, in part due to GluA maturational upregulation of expression. Similarly, as the expression and function of the N-Methyl-D-aspartate (NMDA) subtype of glutamate receptor (GluN) is also developmentally controlled, we examined how early life seizures during the critical period of synaptogenesis could modify GluN development and function. In a postnatal day (P)10 rat model of neonatal seizures, we found that seizures could alter GluN2/3 subunit composition of GluNs and physiological function of synaptic GluNs. In hippocampal slices removed from rats within 48–96 h following seizures, the amplitudes of synaptic GluN-mediated evoked excitatory postsynaptic currents (eEPSCs) were elevated in CA1 pyramidal neurons. Moreover, GluN eEPSCs showed a decreased sensitivity to GluN2B selective antagonists and decreased Mg^2+^ sensitivity at negative holding potentials, indicating a higher proportion of GluN2A and GluN3A subunit function, respectively. These physiological findings were accompanied by a concurrent increase in GluN2A phosphorylation and GluN3A protein. These results suggest that altered GluN function and expression could potentially contribute to future epileptogenesis following neonatal seizures, and may represent potential therapeutic targets for the blockade of future epileptogenesis in the developing brain.

## Introduction

Hypoxic encephalopathy is the most common cause of neonatal seizures, and can lead to the development of long-term epilepsy and cognitive impairments (Jensen et al., 1992; Jensen and Baram, 2000; Rakhade and Jensen, 2009; Bernard and Benke, 2015). Using a rodent hypoxic seizure (HS) model, we have demonstrated that hypoxia at postnatal day 10 (P10) rats causes seizures with later consequences in adulthood, including increased hippocampal (most sensitive to hypoxia; Jensen et al., 1992; Jensen and Baram, 2000) and cortical excitability, epilepsy, and neurobehavioral and cognitive deficits (Rakhade and Jensen, 2009; Zhou et al., 2011; Talos et al., 2012b; Lippman-Bell et al., 2013).

Numerous experimental models reveal that the developing brain is more seizure-prone than the adult (Hellier and Dudek, 2005; Jacobs et al., 2009; Rakhade and Jensen, 2009; Dudek and Staley, 2012). During the critical period of synaptogenesis and plasticity in early brain development, excitatory ionotropic glutamate receptors are overexpressed with unique subunit compositions compared to adulthood (Rakhade and Jensen, 2009). In rodents, sensitivity to HS is highest during the second postnatal week (Jensen et al., 1991; Jensen, 1995), which is the critical period of brain development and synaptogenesis, and hence evaluation of subunit expression may reveal age-specific therapeutic targets for seizure control (Rakhade and Jensen, 2009).

The N-Methyl-D-aspartate (NMDA) subtype of glutamate receptors (GluNs) are heteromeric and composed of different subunits GluN1, GluN2 A-D and GluN3A-B that determine excitatory postsynaptic currents (EPSC) amplitudes and kinetics (Arrigoni and Greene, 2004; Karavanova et al., 2007; Logan et al., 2007; Tong et al., 2008). The GluN2A:2B ratio regulates decay kinetics of GluN EPSCs and efficacy of synaptic plasticity, such as long term potentiation (LTP) and long term depression (LTD). The GluN3A subunits can decrease the Mg^2+^ blockade (Zhou et al., 2009) and Ca^2+^ permeability, and enhance neuronal excitability at resting potentials (Tong et al., 2008; Henson et al., 2010).

Like the other glutamate receptor subtypes, the expression and function of GluN (Jensen et al., 1992; Rakhade and Jensen, 2009; Bernard and Benke, 2015) subunits are developmentally regulated and critical for neuronal migration and synapse formation (Cull-Candy et al., 2001; Zhou et al., 2009; Ohno et al., 2010). In the postnatal developing cortex and hippocampus, synapses express GluNs first without GluAs, which are named as silent synapses and contain GluN1s (Isaac et al., 1997; Zhou et al., 2011). In the rodent cerebrum, overall GluN expression transiently peaks at around P10 (Rakhade and Jensen, 2009; Manning et al., 2011; Talos et al., 2012a; Jantzie et al., 2015). Importantly, there is a developmental switch of GluN2 subunit composition from GluN2B to GluN2A after the second postnatal week in rodent cortex and hippocampus (Kirson and Yaari, 1996; Kumar and Huguenard, 2003; Henson et al., 2008), with similar changes being observed after infancy in humans (Law et al., 2003; Jantzie et al., 2015). Higher GluN2B levels are associated with longer open times and enhanced excitability of the immature brain. In the adult brain, GluN2B appears to be upregulated in epilepsy, as evidenced both from human biopsy tissue with refractory epilepsy or rat epilepsy models (Law et al., 2003; Bustos et al., 2014). Like the GluN2 subunits, the GluN3A subunit is also highly developmentally regulated and transiently overexpressed on hippocampal neurons during the second postnatal week (Wong et al., 2002), and its presence decreases Mg^2+^ blockade (Zhou et al., 2009) and enhances neuronal excitability (Tong et al., 2008). Moreover, interference of GluN3A expression during development can disrupt LTP expression during development (Roberts et al., 2009; Henson et al., 2010).

GluN subunits can be modulated by neuronal activity especially during development (Kerchner and Nicoll, 2008) and GluN2 subunits are phosphorylated by activity-dependent protein kinases (at different serine/tyrosine sites), including the PKA (serines in GluN1, GluN2A and GluN2C), PKC (serines GluN1, GluN2A, GluN2B and GluN2C), CaMKII (serine in GluN2B), Casein Kinase 2 (serine in GluN2B) and Src kinases (at tyrosines in GluN2A, 2B and 2C; Chen and Roche, 2007; Zhou et al., 2007; Zhang et al., 2008; Sanz-Clemente et al., 2010, 2013). This can lead to GluN2 subunit forward trafficking (such as GluN2A at Y1387; Deak et al., 2009; Salter and Kalia, 2004) or endocytosis (such as GluN2B at Y1472; Snyder et al., 2005) and enhance/influence GluN functions (Zahavi et al., 1996; Zheng et al., 1998).

Under conditions of hypoxia and/or ischemia and status epilepticus in the immature brain, GluNs are excessively activated and contribute to seizures and epileptogenesis (Sanchez et al., 2000; Chen and Wasterlain, 2006). In the adult brain, enhanced Ca^2+^ entry through GluNs during seizures can result in altered signal transduction or excitotoxic death (Choi, 1995; Wroge et al., 2012; Clasadonte et al., 2013), which is in part due to altered expression of GluN2A/2B (Gashi et al., 2007; Kristiansen et al., 2010). In this study, we hypothesized that early life seizures could alter synaptic GluN-mediated eEPSC properties and subunit-specific pharmacology, as well as different subunit expression in the developing CA1 pyramidal neurons from P10 to P17. We found that HS increased GluN-eEPSC amplitudes in CA1 pyramidal neurons at 48–96 h following HS at P10, which was mediated by an increased GluN2A phosphorylation and a slightly increased GluN3A subunit expression and function. In addition, enhanced GluN eEPSCs following HS were less sensitive to Mg^2+^ blockade, allowing GluN receptor activation at resting membrane potentials. These alterations in GluN receptors may contribute to later in life spontaneous seizures and epileptogenesis following early life HS.

## Materials and Methods

### Animals

Male Long-Evans hooded rats (Charles River Laboratories, Wilmington, MA, USA) were used in the study. HS at P10 were induced as previously (Rakhade et al., 2008; Zhou et al., 2011). Briefly, HS were induced in P10 rat pups by lowering the oxygen concentration for 15 min (7% for 8 min, 5% for 6 min, and 4% for 1 min). Behavioral seizures, such as head bobbing, wet dog shakes, and tonic-clonic movements, were scored for each animal during 15 min hypoxia. Only rats showing more than five behavioral seizures were included for further analysis. Littermate control pups went through the same procedure with room air. Body temperature was monitored and kept between 32–34°C. All pups were returned to their dams within an hour after the experiment. All procedures were approved by and in accordance with the guidelines of the Animal Care and Use Committee at Boston Children’s Hospital (Boston, MA, USA). All efforts were made to minimize animal suffering and the number of animals used.

### Hippocampal Slice Preparation

Hippocampal slices were prepared as previously described (Zhou et al., 2011). Rat pups were decapitated at 1, 24, 48, 96 h and 1 week post-HS. Littermate rats without hypoxic exposure at same ages were used as normoxic controls. Brains were rapidly dissected from the skull and placed for sectioning in ice-cooled cutting solution containing (mM): 210 Sucrose, 2.5 KCl, 1.02 NaH_2_PO_4_, 0.5 CaCl_2_, 10 MgSO_4_, 26.19 NaHCO_3_, and 10 D-glucose, pH 7.4 bubbled with 95%O_2_/5%CO_2_ at 4°C. Coronal hippocampal slices (300 μm thickness) were sectioned from the middle third of hippocampus with a vibratome (Leica VT1000S, Heidelbeger, Germany) in cutting solution. Slices were incubated in oxygenated artificial cerebrospinal fluid (ACSF) with composition as previously described (Zhou et al., 2011; Sun et al., 2013) and remained at 35°C for 30 min. Then slices were kept at room temperature for at least 1 h before electrophysiological recordings.

### Electrophysiology

Whole-cell patch clamp recordings were made from hippocampal CA1 pyramidal neurons in brain slices using IR-DIC microscope as previously described (Zhou et al., 2009). The patch-pipette internal solution contained (in mM): 110 Cs-methanesulfonate, 10 TEA-Cl, 4 NaCl, 2 MgCl_2_, 10 EGTA, 10 HEPES, 4 ATP-Mg, and 0.3 GTP, pH 7.25, QX-314 (5 mM), phosphocreatine (7 mM) and creatine-phosphokinase (17 unit/ml). Filled electrodes had resistances of 4–6 MΩ. AMPA/KA receptors and γ-aminobutyric acid (GABA) receptors were blocked with NBQX (20 μM) and picrotoxin (60 μM) for all recordings unless otherwise specified. Evoked (e) NMDA-mediated EPSCs were recorded at a holding potential of +40 mV, except that for generating I-V curves for GluN-eEPSCs, cells were held from −80 mV to +50 mV. The Schaffer collaterals from CA3 to CA1 were stimulated at 30 s intervals to obtain stimulus response curve (0.3 ms in duration). The intensity to evoke 60–70% maximal response was used unless otherwise specified. All recordings were performed at room temperature (22–24°C).

Data were collected using an Axopatch200B amplifier (Molecular Devices Inc., Union City, CA, USA) and Clampex 9.2 software (Molecular Devices Inc., Union City, CA, USA) with compensation for series resistance (70%) and cell capacitance, filtered at 2 kHz, and digitized at 20 kHz using a Digidata 1320A analog to digital converter (Molecular Devices Inc., Union City, CA, USA).

### Western Blots

HS and littermate control rat pups were euthanized at 48–96 h after seizures (*n* = 10–20/group) and western blot analysis of micro-dissected CA1 was performed with modification of our previously published protocol (Talos et al., 2012b). Briefly, hippocampal tissue was removed, stretched along the septotemporal axis, and cut into 1 mm sections along the perpendicular axis using a tissue slicer. Sections were placed in chilled ACSF and the CA1 region was isolated under a dissecting microscope. CA1 tissue was pooled between two animals within each group, flash frozen and processed for membrane protein preparations. Tissue was homogenized in lysis buffer containing a Complete Mini Protease Inhibitor Cocktail Tablet (Roche, Germany) and the phosphatase inhibitors phenylmethanesulfonyl fluoride (1 mM), sodium-orthovanadate (1 mM) and okadaic acid (0.1 mM) to block protease and phosphatase activity. Equal amounts of membrane proteins were electrophoresed on 4–20% Tris-HCl gels (Bio-Rad, Hercules, CA, USA) and then transferred to polyvinylidenedifluoride membranes (Bio-Rad). Immunoblots were incubated with GluN2A (1:500, Millipore, Billerica, MA, USA), GluN2B (1:500, Millipore), GluN2C (1:200, Millipore), GluN2D (1:500, Millipore), phospho-GluN2A (Tyr1387; 1:350, abcam, Cambridge, MA, USA), or phospho-GluN2B (Tyr1472; 1:500, abcam) primary antibodies at 4°C overnight. Membranes were then incubated with the appropriate horseradish peroxidase-conjugated anti-rabbit or anti-mouse IgG secondary antibodies (1:5000, Pierce, Rockford, IL, USA). Protein bands were visualized with enhanced chemiluminescence (Pierce), and measured with the Image Reader LAS-4000 system and Image Gauge v3.0 software (Fujifilm). To control for differences in protein loading, raw values were normalized to corresponding β-actin (Sigma-Aldrich) within each immunoblot. Normalized values for each protein were expressed as a percent of the mean expression level of littermate control tissues. Pups in the different groups for same control or HS treatment were pooled to be averaged.

### Statistics

Data was tested for normality using the Shapiro-Wilk normality test. Statistical significance was assessed using a Student’s *t* test, or a one-way ANOVA test when more than two groups were compared for data with normal distributions, and a Mann-Whitney or Wilcoxon matched-pairs signed rank test for comparisons of data that were not normally distributed *p* < 0.05 was considered statistically significant. All results were expressed as mean ± standard error otherwise specified.

## Results

### HS Cause an Early Transient Increase in GluN-Mediated Evoked EPSC Amplitude

**Figure 1:**
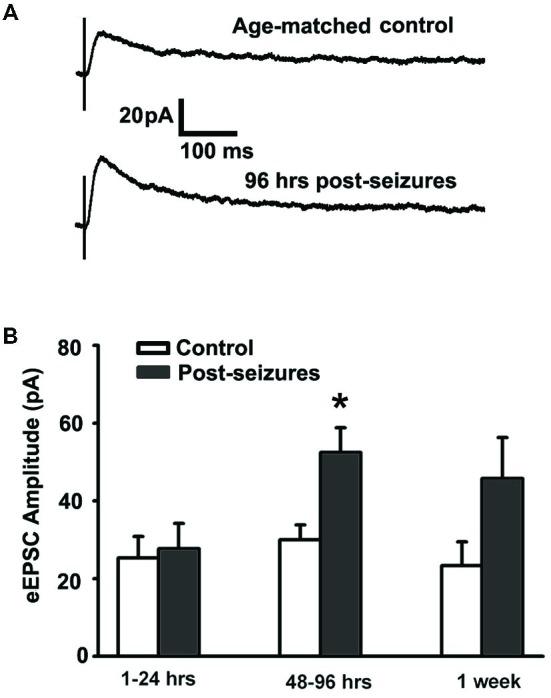
**Hypoxic seizures (HSs) enhance GluN function in hippocampal CA1 pyramidal neurons from P10 to P17. (A)** Representative traces of GluN-mediated eEPSCs, pharmacologically isolated by blocking GluA and GABA_A_ receptors, at a holding potential of +40 mV in hippocampal *ex vivo* slices from control and 96 h post-HS rats. **(B)** GluN-mediated eEPSC amplitude is significantly larger in neurons from 48–96 h post-HS rats (*p* < 0.05, *n* = 11 cells), but not in neurons from 1–24 h post-HS rats (*p* > 0.05, *n* = 11 cells) and 1 week post-HS rats (*p* > 0.05, *n* = 6 cells), compared to littermate control rats (*n* = 6–10 cells). *p < 0.05. Error bars indicate S.E.M.

We examined whether early life HS could alter GluN function as the NMDA subtype of glutamate receptor expression undergoes significant differential regulation during postnatal development (Rakhade and Jensen, 2009). GluN receptors critically contribute to neuronal excitability and seizures (Ghasemi and Schachter, 2011), and in turn they can be altered by neuronal activity (Sanchez et al., 2000; Yashiro and Philpot, 2008; Clasadonte et al., 2013). In this study, we measured evoked GluN-mediated excitatory postsynaptic currents (GluN eEPSCs) from hippocampal CA1pyramidal neurons from 1 h to 7 days post-HS neonatal rats and their littermate controls. GluN eEPSCs in hippocampal CA1 pyramidal neurons showed a peak in amplitude around postnatal (P) 12–14 in both normoxic control rats and post-HS rats (Figures 1A,B). In contrast, GluN eEPSCs showed significantly higher amplitudes in CA1 pyramidal neurons from 48–96 h post-HS rats compared to controls (post-HS 48–96 h: 52.51 ± 6.28pA, *n* = 11 cells *vs* P12–14 normoxic controls: 29.98 ± 3.84pA, *n* = 10 cells, *p* = 0.002, Figures 1A,B), while no significant changes were found at earlier (1–24 h) or later (7 days) time points post HS (*n* = 10–11 cells, *p* > 0.05). These results demonstrate a transient enhancement in GluN eEPSC amplitude following HS in the developing brain, suggesting that function of GluNs during postnatal 48–96 h is sensitive to early hypoxia-induced seizures.

### HS Induced Alterations in GluN2 Function and Expression

**Figure 2:**
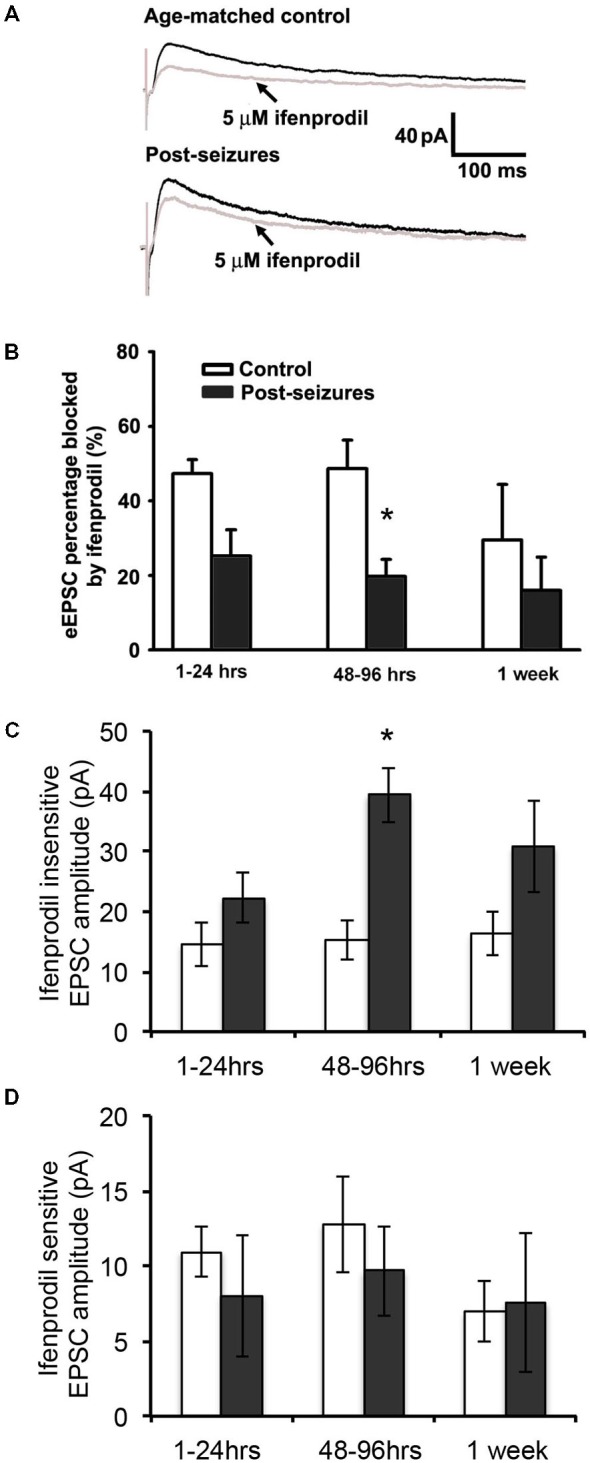
**Hypoxic seizure-induced increases in GluN2A-containing GluN eEPSCs. (A)** Representative GluN eEPSC traces before (black) and after bath application of 5 μM ifenprodil (gray) in hippocampus CA1 pyranmidal neurons at +40 mV holding potential from control and 96 h post-HS rats. **(B)** Group data of ifenprodil sensitivity in CA1 pyramidal neurons in slices from 1–24 h (*n* = 11 cells), 48–96 h (*n* = 11 cells) and 1 week post-HS rats (*n* = 5 cells) and slices from littermate controls (*n* = 6–10 cells). *p < 0.05. Error bars indicate S.E.M. **(C)** Group data of absolute GluN2A-containing GluN eEPSC amplitude (ifenprodil insensitive component) in CA1 pyramidal neurons in slices from 1–24 h (*n* = 11 cells), 48–96 h (*n* = 11 cells) and 1 week post-HS rats (*n* = 5 cells) and slices from littermate controls (*n* = 6–10 cells). *p < 0.05. Error bars indicate S.E.M. **(D)** Group data of absolute GluN2B-containing GluN eEPSC amplitude (ifenprodil sensitive component) in CA1 pyramidal neurons in slices from 1–24 h (*n* = 11 cells), 48–96 h (*n* = 11 cells) and 1 week post-HS rats (*n* = 5 cells) and slices from littermate controls (*n* = 6–10 cells). Error bars indicate S.E.M.

Given that HS-induced changes in GluN eEPSC amplitudes, we next determined whether this was mediated by post-HS alteration in GluN subunits. GluN2A and GluN2B-containing GluNs can be pharmacologically distinguished by sensitivity to blockade by the GluN2B specific blocker ifenprodil (Arrigoni and Greene, 2004). In CA1 pyramidal neurons from hippocampal slices from P12–14 control animals, bath application of ifenprodil (5 μM) significantly decreased GluN eEPSC amplitudes by about 50% (48.62 ± 7.67%, *n* = 11 cells, Figure 2). In contrast, the ifenprodil sensitivity was significantly lower in CA1 pyramidal neurons in slices from post-HS 48–96 h rats (19.74 ± 4.51% reduction, *n* = 10 cells, *p* = 0.009, Figure 2). Moreover, HS subacutely increased the absolute eEPSC amplitude mediated by ifenprodil-insensitive GluN2A-containing GluNs at 48–96 h post-HS (P12–14 control: 15.19 ± 3.18pA; post-HS 48–96 h: 39.37 ± 4.50pA, *n* = 10–11 cells, *p* = 0.0004, Figure 2C). In contrast, at this same time point, there were no changes in the amplitude of ifenprodil-sensitive GluN2B-containing GluNs at 48–96 h post-HS (P12–14 control: 12.75 ± 3.20pA; post-HS 48–96 h: 9.66 ± 3.00pA, *n* = 10–11 cells, *p* = 0.49, Figure 2D). These data suggest that the absolute increases in GluN2A-containing GluN function, without a change in GluN2B function, underlies the sub-acute post-HS enhancement in GluN function. To confirm our findings with ifenprodil, we also tested eEPSC subunit pharmacology with Ro-25–6981, another selective antagonist blocking GluN2B-containing GluNs (Kark et al., 1995; Oren et al., 1995), and similar findings were seen (at P11 normoxia, eEPSC blockade percentage 44.64 ± 7.05%, *n* = 5 by ifenprodil (5 μM) *vs* 36.6 ± 6.34%, *n* = 3 by Ro-25–6981 (0.8 μM); at P11 HS, eEPSC blockade percentage 20.23 ± 10.99%, *n* = 6 by ifenprodil *vs* 18.5 ± 4.36%, *n* = 3 by Ro-25-6981).

**Figure 3:**
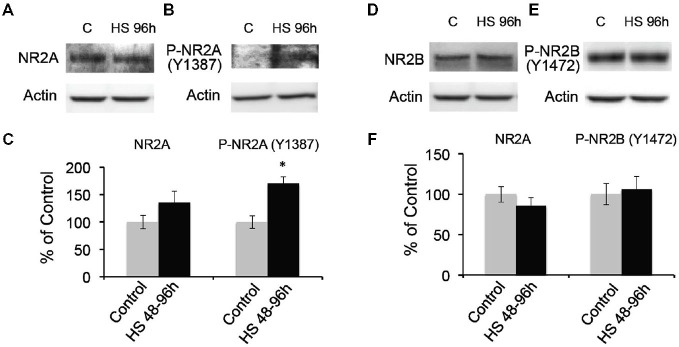
**Hypoxic seizures-induced increases in GluN2A subunit phosphorylation at 48–96 h post-HS. (A–C)** Western blot quantification of total and Phospho-GluN2A subunit (Y1387) in micro-disected hippocampus CA1 from control and 48–96 h post-HS rats (*n* = 20, 20). *p < 0.05. Error bars indicate S.E.M. **(D–F)** Western blot quantification of total and Phospho-GluN2B subunit (Y1472) in micro-dissected hippocampus CA1 from control and 48–96 h post-HS rats (*n* = 10, 10). Error bars indicate S.E.M.

To further confirm the post-HS changes in NMDA receptor GluN2A/B subunit composition, we analyzed the membrane protein expression levels of GluN2A and 2B in the microdissected hippocampal CA1 region from 48–96 h post-HS rats and their littermate controls. We found that GluN2A subunit expression in 1 through to 96 h post-HS rats did not show significant changes compared to their littermate controls (P10–11 controls: 100 ± 16.96%; 1–24 h post-HS rats: 155.22 ± 27.21%, *n* = 20, *p* = 0.09 and P12–14 controls: 100 ± 12.08%; 48–96 h post-HS rats: 135.66 ± 21.06%, *n* = 20, *p* = 0.15, Figure 3A). However, GluN2A phosphorylation at Y1387 in CA1 region at 48–72 h post-HS was significantly increased compared to P12–14 control levels (P12–14 controls: 100 ± 11.31%; 48–96 h post-HS rats: 170.28 ± 12.21%, *n* = 20, *p* = 0.001, Figure 3B), not at 1–24 h (P10–11 controls: 100 ± 14.38%; 1–24 h post-HS rats: 137.98 ± 12.13%, *n* = 20, *p* = 0.07). GluN2A tyrosine (Y1387) phosphorylation functions to incorporate GluN2A subunits into GluNs and enhances GluN function (Chen and Roche, 2007; Zhou et al., 2007; Zhang et al., 2008; Sanz-Clemente et al., 2010, 2013). Our results suggest an increase in GluN2A function at 48–96 h following HS.

In contrast, GluN2B subunit expression in CA1 from 48–96 h post-HS rats did not show significant changes compared to that of their littermate controls from P10 through to P14 (P10–11 controls: 100 ± 10.93%; 1–24 h post-HS rats: 75.09 ± 10.07%, *n* = 20, *p* = 0.10 and P12–14 controls: 100 ± 9.72%; 48–96 h post-HS rats: 86.10 ± 9.89%, *n* = 20, *p* = 0.32, Figure 3C). In addition, phosphorylation of the GluN2B (Y1472) subunit influences the stability of GluN2B in the cell surface membrane (Sanz-Clemente et al., 2010, 2013), and HS did not alter levels of phosphorylation at the Y1472 subunit of GluN2B from P10 through to P14 (P10–11 controls: 100 ± 12.03%; 1–24 h post-HS rats: 97.01 ± 16.44%, *n* = 10, *p* = 0.88 and P12–14 controls: 100 ± 13.22%; 48–96 h post-HS rats: 106.05 ± 15.91%, *n* = 10, *p* = 0.77, Figures 3D–F).

Despite the subacute changes in GluN2 subunits expression, there were no changes in GluN1 subunit expression following HS from P10 through to P14 (at P10–P11 control 100 ± 10.36%, *n* = 10 *vs* 1–24 h post-HS rats: 101.74 ± 13.12%, *n* = 10, *t*-test *p* = 0.918; at P12–14 control 117.03 ± 15.60%, *n* = 10 *vs* 48–96 h post-HS rats: 126.46 ± 17.46%, *n* = 10, *t*-test *p* = 0.691). Taken together, these data indicate that HS in the immature brain induce a specific subacute increase in GluN2A phosphorylation and function.

### HS Decreased Mg^2+^-Sensitivity of GluN-Mediated eEPSCs

**Figure 4:**
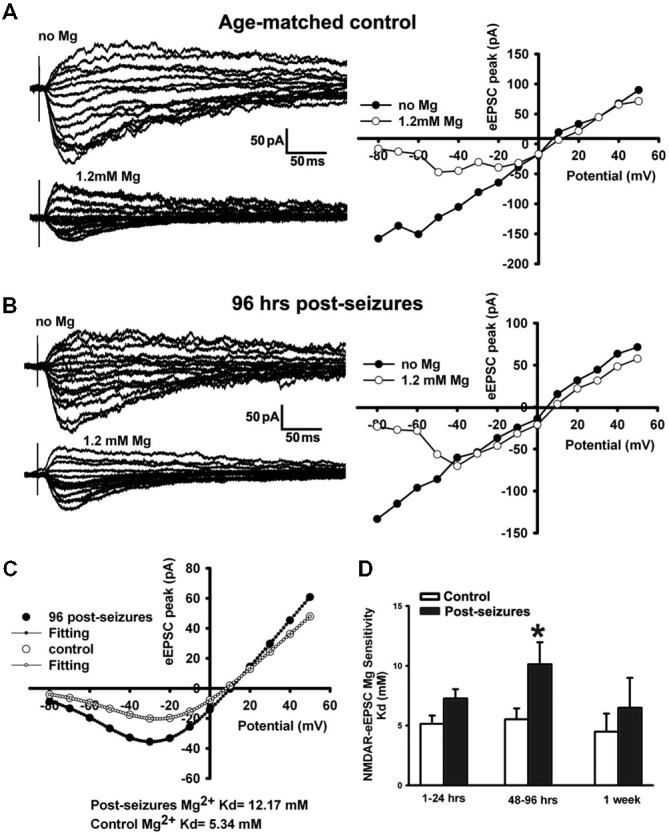
**Reduction of Mg^2+^-sensitivity of GluN eEPSCs at 48–96 h in hippocampal CA1 neurons following HS. (A,B)** Representative traces of evoked GluN EPSCs (left panels) at a holding potential from −80 mV to +50 mV in ACSF with/without Mg^2+^ (1.2 mM) in CA1 pyramidal neurons in slices from control and 96 h post-HS rats. Right panels, corresponding I-V plots of the peak amplitudes of GluN eEPSCs. **(C)** GluN eEPSC I-V curves (with 1.2 mM Mg^2+^ in ACSF) and corresponding fitted Woodhull curves in CA1 pyramidal neurons in slices from control (empty circles) and 96 h post-HS rats (filled circles). **(D)** Group data of fitted Woodhull Kd values in CA1 pyramidal neurons in slices from 1–24 h (*n* = 10 cells), 48–96 h (*n* = 12 cells), 1 week post-HS (*n* = 5 cells) and littermate control rats (*n* = 5–11 cells). ^*^p < 0.05. Error bars indicate S.E.M.

Mg^2+^ sensitivity is one of the critical factors affecting GluN function, and determined by GluN subunit composition (Zhou et al., 2009) as well as post-translational modification (Chen and Huang, 1992). GluN3A subunit confers decreased Mg^2+^ sensitivity and is developmentally upregulated in the first 2 postnatal weeks (Zhou et al., 2009). We thus examined the Mg^2+^-sensitivity of GluN eEPSCs in CA1 pyramidal neurons within postnatal period P12–14 by evoking EPSCs at holding potentials from −80 through +50 mV and measuring linear I-V curves in Mg^2+^-free ACSF. With 1.2 mM Mg^2+^ added into ACSF, inward currents at holding potentials from −80 to 0 mV were suppressed and maximal inward currents appeared at around −30 or −20 mV, exhibiting a typical J-shape I-V curve (Figure 4A). In contrast to age-matched controls (−16.53 ± 4.12 pA, *n* = 7 cells), maximal inward currents at a holding potential of −30 mV in 48–96 h post-HS rats (−36.85 ± 7.31pA, *n* = 7 cells) were significantly larger (Figure 4), suggesting that Mg^2+^ sensitivity of GluNs in post-HS rats is decreased (*t*-test, *p* = 0.032).

To quantify the GluN eEPSC Mg^2+^sensitivity, we applied the Woodhull equation to fit eEPSC I-V curves to derive the Mg^2+^ sensitivity constant Kd (Zhou et al., 2009). Following HS, GluN eEPSCs showed a significantly decreased Mg^2+^ sensitivity in CA1 pyramidal neurons from 48–96 h post-HS rats compared to their littermate controls (48–96 h post-HS Kd: 10.96 ± 1.96mM (smaller sensitivity), *n* = 12 cells; P12–14 control Kd: 5.52 ± 0.92 mM (higher sensitivity), *n* = 11 cells, *p* = 0.024, Figure 4). These results indicate that the Mg^2+^ sensitivity of GluN eEPSCs in post-HS rats was reduced compared to age-matched controls, which would further increase GluN function and contribute to the enhancement of neuronal excitability.

**Figure 5:**
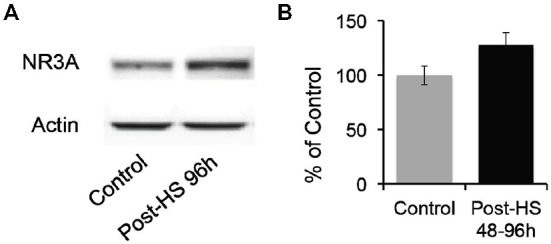
**Hypoxic seizures slightly increase GluN3A subunit expression in hippocampal CA1 neurons. (A)** Representative Western blot of GluN3A subunit in micro-dissected hippocampus CA1 from control and 96 h post-HS rats. **(B)** Western blot quantification of total GluN3A subunit expression in micro-dissected hippocampus CA1 from control and 48–96 h post-HS rats (*n* = 10, 10, *p* = 0.075). Error bars indicate S.E.M.

Given decrease in Mg^2+^ sensitivity, we evaluated whether GluN3A protein expression in the CA1 region of P12–14 rats was altered after seizures at P10 (Figure 5). While there were no significant changes in total protein levels, we found a trend toward increased levels of GluN3A expression in 48–96 h post-HS group compared to their littermate controls (P12–14 controls: 100 ± 8.45%; 48–96 h post-HS rats: 127.41 ± 11.85%, *n* = 10, *p* = 0.075, Figure 5). At earlier time points there was no significant changes at 1–24 h post-HS (P10–11 controls: 100 ± 15.92%; 1–24 h post-HS rats: 118.11 ± 14.90%, *n* = 10, *p* = 0.41).

Because GluN2C/2D containing GluNs can also regulate Mg^2+^ sensitivity, we tested for changes in their expression in the same time window. GluN2C/D subunits were functionally present during these periods (P10–17), indicated by NMDA eEPSC blockade of 60% with 0.8 μM *cis*-PPDA (antagonist for GluN2C/D containing GluNs) and could influence GluN Mg^2+^ sensitivity. However, we did not find any significant difference in GluN2C/D function as indicated by eEPSC blockade with *cis*-PPDA between control and post-HS rats (P10–11 control: 59.47 ± 7.63%, *n* = 3 cells, 1–24 h post-HS: 57.32 ± 11.85% *n* = 4 cells; P12–14 control: 50.03 ± 5.69%, *n* = 8 cells, 48–96 h post-HS: 55.73 ± 4.53%, *n* = 13 cells; P17 control: 59.47 ± 7.63%, *n* = 4 cells, P17 post-HS: 58.65 ± 8.36%, *n* = 5 cells; all *p* > 0.05). These data indicate that HS-induced changes in GluN3A expression and function may mediate the reduction of Mg^2+^ sensitivity of GluNs following HS.

## Discussion

In the present study, we investigated how early life seizures affected NMDA subtype of glutamate receptor (GluN) subunit composition and function in addition to previously identified enhancement in GluA function (Rakhade et al., 2008; Zhou et al., 2011; Sun et al., 2013) in an established neonatal HS model. While increasing evidence supports that GluNs may be critically involved in epileptogenesis in human epilepsy (Endele et al., 2010) and adult animal models of epilepsy (see review in Ghasemi and Schachter, 2011), the role of GluNs in early life epilepsy is still not fully understood (Nairismagi et al., 2006; Swann et al., 2007a). Here, we show that HS enhanced synaptic GluN subunit function and expression in hippocampal CA1 pyramidal neurons. Early post-seizure enhancement of GluN function was in part mediated by increases in GluN2A phosphorylation and GluN3 subunit expression. This overall enhancement of GluN may contribute to enhanced neuronal excitability and epileptogenesis, as well as an alteration of synaptic plasticity seen in this model. These data raise the possibility that modulation of specific GluN subunits may have therapeutic value in preventing cognitive deficits related to seizure-induced alterations in synaptic function.

### Mechanisms Underlying Enhanced GluN eEPSCs and Elevated Neuronal Excitability Following HS

We demonstrated that early-life HS induced an increase in GluN2A and GluN3A function and expression at 48–96 h post-HS in hippocampal CA1 pyramidal neurons. The compositional diversity of GluN2 subunits critically determines the functions of GluNs (Cull-Candy et al., 2001; Chen and Roche, 2007) as well as their roles in the pathophysiology of epilepsy (Ying et al., 2004; Di Maio et al., 2013). GluN2B-containing GluNs have longer channel activating duration and slow deactivation compared to GluNs containing GluN2A subunits (Hilzenrat et al., 1996). Dynamic changes in GluN2A and GluN2B subunits during development result in different combinations of hippocampal NR1/GluN2A, NR1/GluN2B, and NR1/GluN2A/GluN2B receptors (Luo et al., 1997; Hawkins et al., 1999). Normally during development there is a relative abundance of GluN2B subunits, and GluN2A subunits gradually increase during development and are more prevalent in adulthood (Tang et al., 2001; Clayton et al., 2002; Cui et al., 2013). In our model of epilepsy in the immature brain, we found a decreased proportion of GluN2B-containing NMDA eEPSCs in post-HS rats. This was not due to an absolute decrease in GluN2B-containing GluNs, but an increase in the absolute amount of GluN2A-containing receptors likely mediated by the observed increase in GluN2A subunit phosphorylation (Y1387 by Src kinase). The Y1387 site on GluN2A is phosphorylated in an activity dependent manner by Src kinase (Ali and Salter, 2001; Salter and Kalia, 2004) and contributed to synaptic plasticity, yet this is the first documentation of this posttranslational change being mediated by seizures. We also observed a strong trend of increase in GluN3A subunits, which could result in less Mg^2+^ blockade of GluNs at negative holding potentials, increasing the likelihood of activation. The decreased Mg^2+^ blockade was likely specific to increases in GluN3A, as we did not observe significant changes of GluN2C/D containing GluN eEPSCs which are also known to regulate Mg^2+^ sensitivity of GluNs. Taken together, the enhanced GluN function following HS in the immature brain appears to be primarily due to an upregulation of both GluN2A (Src phosphorylation) and GluN3A-containing GluNs. This pattern is distinct from that in the adult brain, where increases of GluN2B-containing GluNs have been reported in adult epileptic tissue and adult rodent models (DeFazio and Hablitz, 2000; Ying et al., 2004; Borbély et al., 2009; Durakoglugil et al., 2009; Sun et al., 2009; Endele et al., 2010; Di Maio et al., 2013).

GluN2A and GluN2B subunit function can be modulated by post-translational phosphorylation. We have previously reported an increase in the activity of several kinases, including PKA, PKC, CamKII and TrkB, during the subacute period following HS (Rakhade et al., 2008; Obeid et al., 2014). Phosphorylation of GluNs can also influence the amplitude of GluNs and Mg^2+^ sensitivity (Chen and Huang, 1992). Phosphorylation of GluN2A and GluN2B can influence their membrane surface expression or endocytosis (Chen and Roche, 2007), which can directly contribute to GluN current amplitudes and change neuronal excitability. Such post-translational modification has been reported to occur in models of synaptic plasticity, but this is the first report of such a change in the epileptic immature brain.

There were no alterations in GluN1 total subunit protein following HS in the current study. However, this result is consistent with our previous immunocyto-chemical finding in silent synapse study (Zhou et al., 2011), in which synaptic GluN1 labeling is not altered at post-HS 48 h, although the colocalization with GluA1 is increased. The present study builds upon the fact that in addition to an unsilencing of GluN receptors by seizures, the kinetics of the NMDA receptor is further modified by GluN2A and GluN3A alterations.

### Enhanced GluN2A Function by Phosphorylation would Facilitate its Role of Pro-Epileptogenesis Following HS

The GluN2A subunit has been previously shown to increase due to epileptic activity in animal seizure models (Hellier et al., 2009; Gibbs et al., 2011; Reid et al., 2012; see Chen et al., 2007 for a reduction of GluN2A following flurothyl-induced recurrent neonatal seizures), which may also be associated with mossy fiber sprouting (Swann et al., 2007b). In the present study, we found that the increase in GluN2A subunit phosphorylation accounts for HS-induced reduction of ifenprodil sensitivity, rather than a change in the absolute levels of GluN2B. GluN2A subunit phosphorylation at Y1387 is mediated by Src kinase, which can decrease tonic zinc inhibition of GluNs at synapses (Eaton et al., 1995; Perouansky et al., 1996; Zahavi et al., 1996). This may account for enhancement of GluN2A containing GluN eEPSCs without an observed increase of GluN2A expression. Hamada et al. (2014) clearly showed that one main function of NR2A subunit is to regulate AMPARs into synaptic sites during neonatal period. Gray et al. (2011) showed that deletion of GluN2A subunit will decrease synaptic strength. These would imply that increase of GluN2A function due to phosphorylation in our current study would lead to higher neuronal excitability through enhanced GluA function, consistent with our previous findings (Rakhade et al., 2008; Zhou et al., 2011).

### GluN3A may Contribute to the Post-HS Neuronal Excitability

GluN3A containing GluNs exert critical effects on GluN receptor function and neuronal excitability in the immature brain (Henson et al., 2010). We showed that early life seizures decreased GluN Mg^2+^ sensitivity, suggestive of a increase in GluN3A protein expression in CA1 neurons at 48–96 h post-HS, although we only were able to show a trend in protein expression (due to commercial antibody unavailability of potential GluN3A phosphorylation (Henson et al., 2010), we can not exclude that the possible elevated/altered GluN3A phosphorylation may enhance GluN3A functions following HS). The GluN3A subunit is most highly expressed during the early developmental period (Zhou et al., 2009; Henson et al., 2010; Sucher et al., 2010). One of most prominent features of GluN3A-containing GluNs is almost complete insensitivity to Mg^2+^ blockade at a hyperpolarized potential (Sasaki et al., 2002; Henson et al., 2010). Incorporation of more GluN3A subunits into GluNs has been shown to increase the currents at resting potentials (Zhou et al., 2009), which would result in elevated neuronal excitability (Espinosa and Kavalali, 2009) and seizures which is implied in human epileptic patients with mutant GluNs with decreased Mg^2+^ blockade and less Ca^2+^ permeability (Endele et al., 2010). In our study, the increase of eEPSC amplitudes at negative potentials is likely due to HS-induced decreases in Mg^2+^ blockade of GluNs at negative potentials (Zhou et al., 2009), which allows GluNs to be active at negative membrane potentials and makes CA1 pyramidal neurons more excitable at resting membrane potential. While GluN2C/2D subunits can also regulate Mg^2+^ sensitivity, we were able to rule out a contribution due to these subunits as there was no differential sensitivity to the GluN2C/D blocker cis-PPDA on GluN-eEPSCs between normoxic control and post-HS group. Thus, in our study, HS-induced a strong trend of increases in the expression of GluN3A subunit may be more likely to cause of the decreased Mg^2+^ sensitivity of GluNs. While the GluN3A total protein expression is not significantly altered, the fact that our results show that GluN2C or GluN2D are unaltered, it is possible that future studies can examine post-translational modifications as antibodies become available.

### HS-Induced Alteration of Critical Elements of GluNs that Regulate Synaptic Plasticity Following HS

GluNs have been demonstrated to be involved in synaptic plasticity in previous studies (Flint et al., 1997; Morishita et al., 2007; Yashiro and Philpot, 2008; Cho et al., 2009). The subunit composition of GluNs has been shown to be involved in LTP or LTD induction. In our study, more GluN2A containing GluNs seem to be expressed following HS compared with GluN2B containing GluNs, which overlaps with the time window (P12–14) when LTP expression is occluded (Zhou et al., 2011). Specifically, more GluN2A (or less GluN2B) containing GluNs decreases the amplitude of LTP (Massey et al., 2004; Bartlett et al., 2007; Cho et al., 2009). The increase of GluN2A containing GluNs could induce rapid kinetics of GluNs, and therefore less Ca^2+^ influx, which may prevent the trafficking GluAs to synapses and thus contribute to LTP occlusion following neonatal seizures (Gambrill and Barria, 2011; Zhou et al., 2011). In addition to GluN2A, incorporation of GluN3A subunit into GluNs decreases GluNs’ Ca^2+^ permeability and Mg^2+^ blockade, which could also result in the deficit of synaptic plasticity (Roberts et al., 2009; Larsen et al., 2011). Thus, increases of both GluN2A and GluN3A containing GluNs following neonatal seizures could reduce neuronal capability to induce LTP, which is consistent with our previous finding of LTP occlusion at 48–72 h post neonatal seizures (Zhou et al., 2011).

In summary, this study has provided the evidence that GluNs are involved in early-life HSs and very likely contribute to neuronal hyper-excitability and later life epileptogenesis. This finding complements our previous findings on critical roles of HS-induced enhancement on GluA expression and function in early life epilepsy (Rakhade et al., 2008; Zhou et al., 2011; Lippman-Bell et al., 2013), and provides an additional mechanism whereby seizures in the immature brain can cause a dysmature state. These data suggest that modulation of specific GluN subunits may represent a new potential therapeutic target in preventing cognitive deficits related to seizure-induced alterations in synaptic function and impairment in synaptic plasticity.

## Conflict of Interest Statement

The authors declare that the research was conducted in the absence of any commercial or financial relationships that could be construed as a potential conflict of interest.
